# Differences and Similarities in the Peptide Profile of Preterm and Term Mother’s Milk, and Preterm and Term Infant Gastric Samples

**DOI:** 10.3390/nu12092825

**Published:** 2020-09-15

**Authors:** Søren D. Nielsen, Robert L. Beverly, Mark A. Underwood, David C. Dallas

**Affiliations:** 1 Nutrition Program, School of Biological and Population Health Sciences, College of Public Health and Human Sciences, Oregon State University, Corvallis, OR 97331, USA; sodn@food.au.dk (S.D.N.); beverlyr@oregonstate.edu (R.L.B.); 2 Department of Food Science, Faculty of Technical Sciences, Aarhus University, 8200 Aarhus, Denmark; 3 Department of Pediatrics, University of California, Sacramento, CA 95817, USA; munderwood@ucdavis.edu

**Keywords:** premature infant, term infant, peptidomics, nutrition, human milk, bioactive

## Abstract

Our previous studies revealed that milk proteases begin to hydrolyze proteins in the mammary gland and that proteolytic digestion continues within the infant stomach. No research has measured how the release of milk peptides differs between the gastric aspirates of term and premature infants. This study examined the presence of milk peptides in milk and gastric samples from term and preterm infants using an Orbitrap Fusion Lumos mass spectrometer. Samples were collected from nine preterm-delivering and four term-delivering mother–infant pairs. Our study reveals an increased count and ion abundance of peptides and decreased peptide length from mother’s milk to the infant stomach, confirming that additional break-down of the milk proteins occurred in both preterm and term infants’ stomachs. Protein digestion occurred at a higher level in the gastric contents of term infants than in gastric contents of preterm infants. An amino acid cleavage site-based enzyme analysis suggested that the observed higher proteolysis in the term infants was due to higher pepsin/cathepsin D activity in the stomach. Additionally, there was a higher quantity of antimicrobial peptides in term infant gastric contents than in those of preterm infants, which could indicate that preterm infants benefit less from bioactive peptides in the gut.

## 1. Introduction

Preterm birth, defined as birth prior to 37 weeks of gestational age (GA), occurs in nearly one in every ten births in the United States [1]. Despite advances in medicine that have increased the survival of preterm infants in recent decades, these infants remain at increased risk for morbidities related to underdevelopment compared with their term counterparts [2,3].

Preterm infants have underdeveloped gastrointestinal (GI) systems that may impede their ability to adequately break down the complex macronutrients provided by mother’s milk, which could be partially responsible for their increased risk of growth inadequacy and developmental issues. At birth, the means by which these infants acquire nutrients shifts from the direct supply of sugars, amino acids, and free fatty acids via the mother’s placenta to ingestion of milk’s carbohydrates, lipids, and proteins, which must be digested and absorbed. Protein digestion in the stomach is initiated by the secretion of gastric acid and pepsin, which denature and break down the primary structure of the food proteins. Preterm infants produce less gastric acid in the first week of life [4] and less gastric pepsin than term infants [5,6], likely resulting in a lower capacity to digest milk proteins in the stomach. Recent research demonstrated that preterm infant gastric samples contain lower protease activity and less total proteolysis of milk proteins than term infant gastric samples [7].

Lower gastric protein digestion may limit the amount of amino acids available for absorption in the small intestine. Furthermore, altered protein digestion due to infant prematurity could lead to differential release of specific peptide fragments from mothers’ milk proteins. Peptidomics is one technique commonly used to measure the digestion of milk proteins. Peptides released during in vitro digestions of bovine and human milk proteins have been analyzed several times [8,9], but in vitro digests often fail to adequately represent the immature infant GI system [10,11]. Piglet models have been used to measure formula digestion in the stomach, jejunum, and ileum [12], which is more representative of the infant GI system, but infant studies remain the gold standard. In preterm infants, it was shown that milk peptides increase in count and abundance from human milk to the infant stomach and increase in the stomach over time [13,14,15]. However, comparisons of preterm and term infants are sparse, even using in vitro and animal models. The only comparisons of total milk peptide release between preterm and term infants were performed on human milk and infant stool [16,17], and only milk immunoglobulin peptides have been compared in preterm and term gastric samples [18].

Differences in milk protein digestion are significant beyond the ability to release amino acids for absorption. Milk proteins contain an array of encrypted fragments that are released upon partial proteolysis; these fragments have an array of bioactive functions, including antimicrobial [19], immunomodulatory [20], antihypertensive [21], opioid [22], and intestinal wound healing [23,24]. Several of these peptides have been identified in human milk samples and infant digesta [13]. Lower release of these bioactive peptides in preterm compared with term infants could contribute to preterm infants’ increased risk of negative health outcomes.

The purpose of this study was to compare the peptide profiles of the milks of preterm- and term-delivering mothers and the gastric samples of term and preterm infants. Although previous studies have identified that preterm infant stomachs may have reduced protein digestion capacity, the impact of this immaturity on the release of bioactive peptides from human milk remains unknown. The peptidomic comparisons in this study will lead to a deeper understanding of the specific limitations in the release of bioactive peptides from milk protein in the preterm infant stomach to enable future research examining the biological relevance of these differences to preterm infant health outcomes.

## 2. Materials and Methods

### 2.1. Participants and Sample Collection

This study was approved by the institutional review boards of the University of California, Davis (UC Davis) and Oregon State University, Corvallis, OR, USA. Human milk and infant gastric samples were collected from nine preterm-delivering mother–infant pairs ranging in GA at birth from 24 to 32 weeks and birth weight from 620–1610 g during 8–41 days of postnatal age at the UC Davis Children’s Hospital Neonatal Intensive Care Unit in Sacramento, CA. Human milk and infant gastric samples were also collected from four term infants with a GA of 38–40 weeks and birth weight from 3360–3837 g during 14–42 days of postnatal age. Infants enrolled in this observational study each had health issues but no overt GI tract issues. The infants’ conditions precluded normal breastfeeding; therefore, a nasogastric feeding tube was placed into each infant. Breast milk samples (unfortified) were collected by pumping on-site or at home with clean electric breast pumps into sterile plastic containers and stored immediately at −20 °C. The breast was cleaned with water on a washcloth (no soap or alcohol) before pumping. The human milk feedings (fortified with bovine-based human milk fortifier or high-calorie infant formula for preterm and term infants, respectively) were delivered via the nasogastric tubes over 30 min. Two hours after the initiation of feeding, a fraction of each infant’s gastric contents was collected in a 3 mL syringe back through the feeding tube via suction. Gastric samples were placed into sterile plastic vials and stored immediately at −20 °C. Human milk and gastric samples were transported to Oregon State University on dry ice and stored at −80 °C.

### 2.2. Sample Preparation

Human milk and gastric samples were thawed at 4 °C. Afterwards, samples were centrifuged at 4226× *g* for 10 min at 4 °C, placed on ice, and the infranate was collected by pipette from below the upper fat layer. Milk proteins were then precipitated from the skimmed milk by addition of 400 µL of 240 g/L trichloroacetic acid. After mixing for 10 s with a vortex mixer, the samples were centrifuged at 4000× *g* for 10 min at 4 °C and 600 µL of the supernatant containing the peptides was collected. Trichloroacetic acid, salts, oligosaccharides, and lactose were removed from the peptide solution and peptides were extracted using C18 reverse-phase preparative chromatography 96-well plates (Glygen, Columbia, MD, USA) as previously described [25]. The sample eluate was transferred to a new labeled tube after each centrifugation. The peptide solutions were frozen at −80 °C and lyophilized using a freeze-dry system (Labconco FreeZone 4.5 L, Kansas City, MO, USA). After drying, the samples were rehydrated in 0.1% formic acid in water to their initial amount.

### 2.3. Liquid Chromatography (LC) Nano-Electrospray Ionization Mass Spectrometry

The samples were loaded onto a 180 µm × 20 mm, 5 µm bead 2G nanoAcquity UPLC trap column (reverse phase) for enrichment and online desalting, and then onto a 100 µm × 100 mm, 1.7 µm bead Acquity UPLC Peptide BEH C18 column (Waters Corporation, Milford, MA, USA) for analytical separation on a nanoAcquity UPLC (Waters Corporation) directly connected with a nanospray source. The LC eluent was set up as previously described [25].

The mass spectrometry (MS) instrument used was a Thermo Scientific Orbitrap Fusion Lumos. Spectra were collected with positive-ionization mode with an electrospray voltage of 2400 V. The MS scan range was 400–1500 m/z at a resolution of 120 K. The automatic gain control target was set to 4.0 ×10^5^, with a maximum injection time of 50 ms. The fragmentation mode was set to collision-induced dissociation, and the collision energy was 35%. The MS cycle time was set to 3 s, with data-dependent analysis and automated precursor peak selection. Precursors were excluded (within 10 ppm mass error) after one fragmentation for 60 s. Precursors were selected for fragmentation based on the following criteria: most intense peaks, ion-intensity threshold 5.0 × 10^3^, and charge state 2–7. Fragments were detected with the ion trap with an automatic scan range.

The collected spectra were analyzed by database searching in Thermo Proteome Discoverer (v2.1.0.81) using an in-house human milk protein sequence database. Potential modifications allowed included phosphorylation of serine and threonine and oxidation of methionine. Only peptides identified with high confidence were included (*p* < 0.01), and peptide sequences with multiple modifications were grouped into a single peptide for counts. Counts measured the number of unique peptide sequences identified in a sample. Abundance measured the area under the curve of the eluted peak (ion intensity).

### 2.4. Protein Concentration

The protein concentration in human milk and gastric samples prior to sample preparation were measured with the bicinchoninic acid protein assay.

### 2.5. Bioactive Peptide Prediction

Peptides identified in milk and gastric samples were examined for homology with literature-identified bioactive peptides using our Milk Bioactive Peptide Database (MBPDB, http://mbpdb.nws.oregonstate.edu/) [26]. The MBPDB is a comprehensive database covering all known milk bioactive peptides. The search was performed as a sequence search that searches for bioactive peptides matching the input peptide sequence. The “similarity threshold” was set to 80%. In the result file, “Get extra output” was included to obtain the specific percentage similarity between the query sequence and the database sequence.

### 2.6. Statistics

Differences in preterm and term overall peptide intensity and count as well as peptide count per protein were determined using an analysis of variance with Tukey’s HSD post hoc test in the statistical program RStudio.

For protein differences, data were analyzed with Perseus v.1.6.1.1 (Planegg, Germany). Once data were loaded into the software, they were log2-transformed and grouped into preterm milk, term milk, preterm gastric, and term gastric. Data were filtered by rows based on valid values for occurrence in ≥75% of samples in at least one group. A volcano plot was constructed using the volcano plot function in Perseus based on log2 fold change on the x-axis and −log(*p*-value) on the y-axis. An ANOVA test was performed in Perseus to determine whether there were significant differences between the abundance of amino acids positioned at the P1 or P1′ position of the enzymatic cleavage sites and between the abundance of bioactive peptides in preterm and term mother’s milk and the infant gastric samples. Two sample *t-*tests were used to compare each amino acid in the heat map. Throughout the paper, significance was defined as *p* < 0.05.

## 3. Results

### 3.1. Demographic Information

Demographic details for the preterm and term mother–infant pairs are presented in Table 1. Human milk peptidomic analysis was performed on unfortified milk samples. The gastric aspirates from the preterm infants were obtained following feeding of human milk that was fortified with bovine-based human milk fortifier. The gastric aspirates from the term infants were obtained following fortification of human milk with high-calorie infant formula. 

### 3.2. Protein Concentration and pH

The protein concentration of the unfortified preterm human milk was 16.1 ± 0.5 mg/mL and that of term human milk was 14.6 ± 1.9 mg/mL. The gastric aspirates collected two hours after feeding contained 17.07 ± 1.5 mg/mL and 17.9 ± 4.5 mg/mL for preterm and term infants, respectively. The pH of the preterm human milk was 6.29 ± 0.1, and the pH of the term human milk was 6.52 ± 0.11. The pH of the gastric aspirates was 4.41 ± 0.37 in preterm infants and 4.51 ± 0.24 in term infants.

### 3.3. Total Peptide Identification

**Figure 1:**
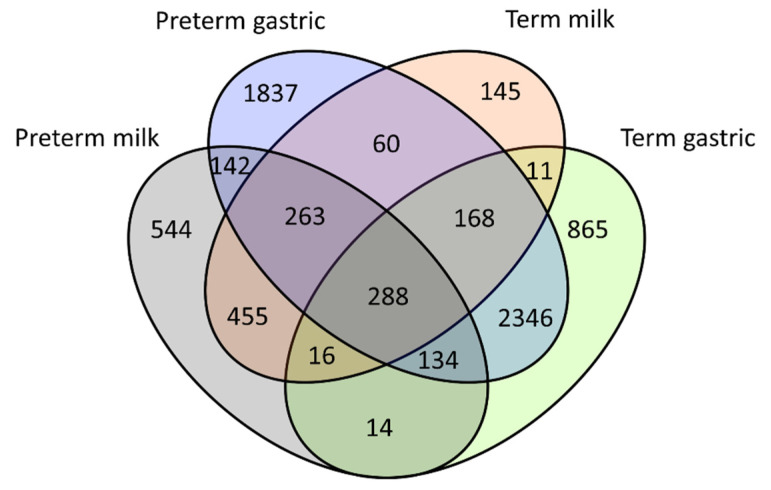
Venn diagram showing number of peptides identified in each sample group and their overlaps with other samples groups.

This study compared the number of peptides released from the proteins present in term and preterm mothers’ milk and those released in the preterm and term infant stomachs. A total of 2240 unique peptides were identified in the 13 human milk samples from 79 milk proteins. Of these, 834 were found in preterm milk and not term milk, while 384 were found in term mothers’ milk and not preterm mothers’ milk (Figure 1). The remaining 1022 were identified in both term and preterm mothers’ milks (Figure 1). Of the milk peptides, 85 were found in all mothers’ milk samples, and accounted for 70.9 ± 4.4% of the total milk peptide abundance.

A total of 6144 unique peptides were identified in the 13 infant gastric samples deriving from 127 different human milk proteins and 89 different bovine milk proteins. Of these, 2302 were identified in preterm gastric and not term gastric, whereas 906 were identified in term gastric and not preterm gastric (Figure 1). One hundred and twelve peptides were present in all the gastric samples, accounting for 34.3 ± 5.3% of the total gastric peptide abundance. Seven peptides were identified in all milk and gastric samples (human α_s1_-casein R^1^-N^35^ and R^10^-N^35^, human β-casein E^2^-K^18^, L^130^-T^145^, L^188^-V^211^, L^189^-V^211^, P^201^-V^211^).

**Figure 2:**
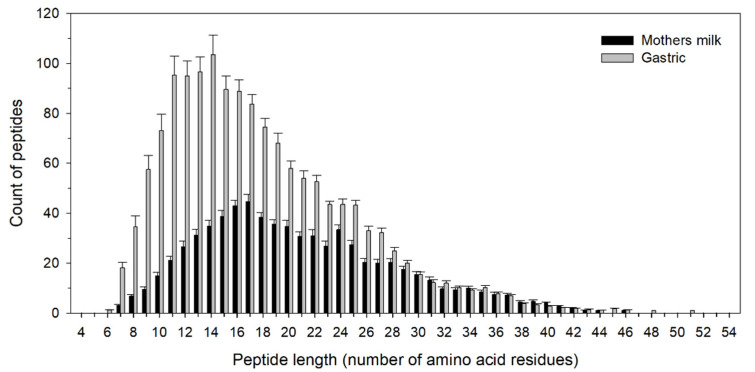
Distribution of peptide length in milk and gastric samples. Data are presented as mean ± standard error, *n =* 13.

The amino acid length of the identified peptides was investigated in milk and gastric samples. The combined data for preterm and term infants showed an increased number of short peptides in the gastric compared with the milk samples (Figure 2).

### 3.4. Comparison of Peptide Profile between Preterm and Term Mothers’ Milk and Infant Gastric Contents

**Figure 3:**
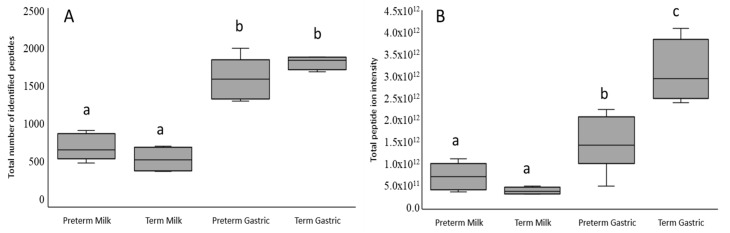
Differences in total number of peptides identified in mothers’ milk and infant gastric. Total count (**A**) and abundance (**B**) of peptides identified in preterm and term infant gastric samples (human milk fortified with bovine-based human milk fortifier or high-calorie infant formula, respectively) and their mothers’ milk (unfortified). Different letters indicate significantly different values (*p* < 0.05).

On average, 725.0 ± 54.3 and 595.0 ± 81.5 peptides were identified in the preterm and term mothers’ milk samples (*p* = 0.23), respectively (Figure 3A). The average total peptide abundance tended to be higher in preterm mothers’ milk than in term mothers’ milk (*p* = 0.09). The majority of peptides in mother’s milk derived from, in order of peptide count pooled across term and preterm, human β-casein, human osteopontin, human polymeric immunoglobulin receptor, human α_s1_-casein, human butyrophilin subfamily 1 member A1, human bile salt-activated lipase, human κ-casein, and human mucin-1. The count of peptides derived from α_s1_-casein and polymeric immunoglobulin receptor was higher in the preterm mothers’ milk than in term mothers’ milk (Table 2).

On average, 1698.7 ± 113.4 and 1816.3 ± 43.5 peptides were identified in preterm and term infant gastric samples (Figure 3B) (*p* = 0.35), which was for both preterm and term infants significantly higher than in the milk samples (*p* < 0.001). The finding that the number of peptides present in the stomach was higher than in milk conforms with a previous study [15]. The average total peptide abundance was significantly higher in term infant gastric contents compared with preterm infant gastric contents (*p* < 0.001). The total abundance of peptides increased significantly from mothers’ milk to infant gastric contents, in both preterm and term infants.

The majority of peptides derived from—in order of peptide count pooled across term and preterm gastric samples—human β-casein, bovine β-casein, bovine α_s1_-casein, bovine κ-casein, human α_s1_-casein, bovine β-lactoglobulin, human osteopontin, human polymeric immunoglobulin receptor, human lactoferrin, bovine α_s2_-casein (Table 2). The count of peptides identified from lactoferrin was significantly higher in the preterm infant stomach compared with the term infant stomach.

### 3.5. Individual Protein Digestion

**Figure 4:**
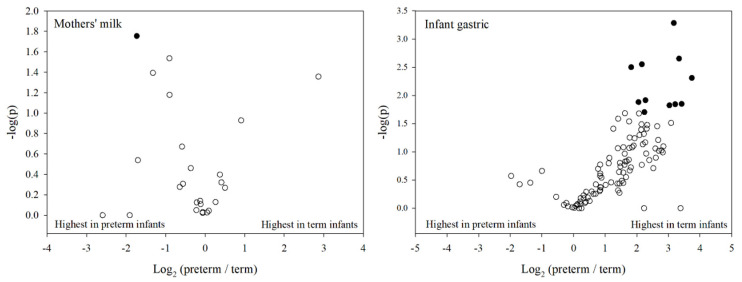
Volcano plots depicting fold change in total peptide intensity from individual proteins (*x-*axis, logarithmic) and *p*-value (y-axis, logarithmic) between term and preterm infant milk and gastric samples. Filled circles indicate the protein was significantly different between preterm and term infants (*p* < 0.05), whereas the hollow circles are non-significant.

To investigate from which specific proteins the release of peptides was significantly different between preterm and term infant samples, we constructed a volcano plot showing the total abundance of peptides from each protein in either preterm or term milk or gastric samples (Figure 4). We included only proteins that were identified in at least 75% of samples in either preterm or term milk or gastric samples. Twenty-five proteins in milk samples and 106 proteins in the gastric samples met these criteria. In milk, peptides from 17 proteins (68% of the proteins meeting the criteria) were more abundant in preterm mothers’ milk than in term mothers’ milk, but only peptides from α_s1_-casein were significantly more abundant in preterm mothers’ milk compared with term mothers’ milk. In gastric samples, peptides from 97 (92%) proteins trended towards greater abundance in term infants than in preterm infants. The peptide abundances of eleven of these proteins were significantly higher in term infant gastric samples than in preterm infant gastric samples. The eleven significantly different proteins were human Ig gamma-1, human gelsolin, human apolipoprotein A-I 1, human α-2-HS-glycoprotein, human α-enolase, human fibrinogen, bovine osteopontin, bovine heat shock 70kDa protein 1A, human serum albumin, human peptidyl-prolyl cis-trans isomerase A, and human apolipoprotein A-II.

**Figure 5:**
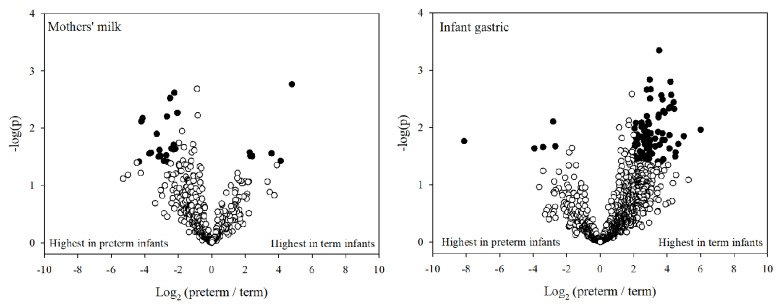
Volcano plots depicting fold change in individual peptide intensity (*x-*axis, logarithmic) and *p-*value (*y*-axis, logarithmic) between term and preterm infant milk and gastric samples. Filled circles indicate the peptide was significantly different between preterm and term infants (*p* < 0.05), whereas the hollow circles are non-significant.

For comparison between preterm and term infants at a peptide level, we excluded all peptides that were present in less than 75% of samples in either preterm or term milk or gastric samples. After this filtering, 1408 peptides remained, of which 439 peptides were in the human milk samples and 1049 were in the infant gastric samples. Although this filtering step largely reduced the number of peptides, the remaining peptides accounted for an average of 88% of the total ion intensity identified before filtering. The difference and fold change between preterm and term infants of these peptides are shown in Figure 5. The majority of peptides (279 out of 439) showed a trend towards higher abundance in preterm mothers’ milk compared with term mothers’ milk, of these 19 were significantly more abundant. In the infant gastric contents, the majority of peptides (770 out of 1049) trended towards greater abundance in term infants compared with preterm infants, of which 80 were significantly higher.

**Figure 6:**
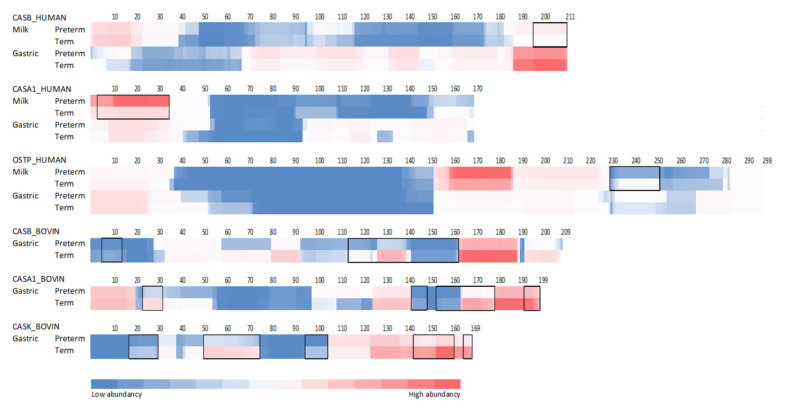
Abundance of peptides identified in mother’s milk and gastric samples from term and preterm infants mapped to the sequence of human and bovine β-casein (CASB), osteopontin (OSTP), α_s1_-casein (CASA1), and κ-casein (CASK). Areas with black borders mark significant difference (*p* < 0.05) between ion intensity of peptides at each amino acid site.

Heatmap analysis of peptide release across their parent protein sequences demonstrates that peptides were released at similar regions across α_s1_-casein, β-casein, and osteopontin sequences between preterm and term infants in both milk samples and gastric samples (Figure 6). Based on ion intensity, the quantity of peptides from the N-terminal of human β-CN was significantly higher in term milk compared with preterm milk. The ion intensity of peptides from the C-terminal of human α_s1_-casein was significantly higher in preterm milk than in term milk. Amino acid residues 231–250 of human osteopontin were significantly higher in term milk compared with preterm milk. 

In gastric samples, there was a significantly higher ion intensity of peptides from human osteopontin at amino acid residues position 215–252. Neither human β-casein nor human α_s1_-casein gastric peptide intensities differed between preterm and term infants. For bovine β-casein and bovine α_s1_-casein, the ion intensity of peptides deriving from their C-termini were significantly higher in the gastric contents of term infants compared with preterm infants. The ion intensity of peptides deriving from several areas of the bovine κ-CN were higher in the gastric contents of term infants compared with that of preterm infants (Figure 6).

### 3.6. Analysis of Proteolytic Cleavage Sites

We examined the amino acids located at P1 and P1′ of the C- and N-termini of the identified peptides to link cleavage patterns with known proteases. P1 is the amino acid positioned just prior to the enzymatic cleavage site and P1′ is the amino acid positioned just after the enzymatic cleavage site in the protein sequence. As some of the enzymes present and active in milk and infant gastric contents have high specificity for certain amino acids at either the P1 or P1′ position, we used the cleavage sites to identify which specific enzymatic activity caused the release of identified peptides.

In human milk, most of the cleavages in milk proteins were after an arginine or a lysine, which matches the cleavage specificity of plasmin, kallikrein 6 and 11, and thrombin present in human milk [27]. 

**Figure 7:**
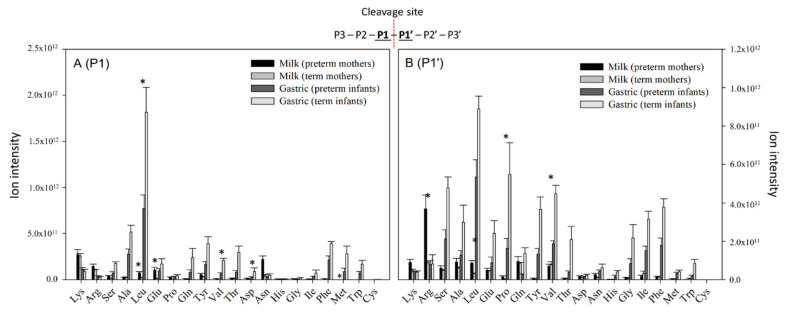
Total ion intensity of peptides distributed according to their P1 (**A**) and P1′ (**B**) cleavage site amino acid in term and preterm infants’ mothers’ milk and their gastric samples. Asterisk marks significant difference between preterm and term infants. P3–P3’ are the amino acids positioned around the cleavage site.

In the gastric samples of preterm and term infants, most cleavages occurred after a leucine, which matches the cleavage specificity of pepsin and cathepsin D (Figure 7). Peptides resulting from cleavage after a leucine were significantly higher in term infants compared with preterm infants, indicating higher activity of pepsin and cathepsin D in term infants. In gastric samples, peptides with a proline or valine at the P1′ position were also significantly higher in term infants than in preterm infants. No gastric or milk enzyme with specificity for proline or valine at P1′ position is known.

### 3.7. Bioactive Peptide Comparison between Preterm and Term Infants

From the total number of identified peptides in milk and gastric samples, we identified 436 peptides with high similarity to a known bioactive peptide (>80% sequence homology) deriving from bovine milk proteins (207 with angiotensin-converting enzyme (ACE)-inhibitory activity, 135 with antimicrobial activity, 22 with dipeptidyl peptidase IV inhibitory activity, 7 with antioxidant activity, and 65 with other biological functions) and 145 peptides deriving from human milk proteins (54 with ACE-inhibitory activity, 48 with antimicrobial activity, 40 with cell proliferation-stimulatory activity, and 3 with other biological functions). Five peptides found in the milk and 35 peptides found in gastric samples were identical to a known bioactive peptide.

**Figure 8:**
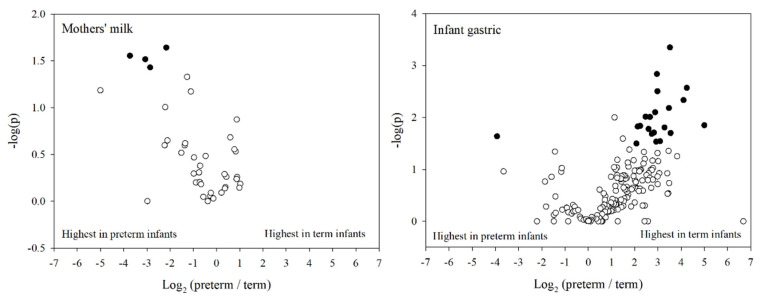
Volcano plots depicting fold change in bioactive peptide intensity (*x-*axis, logarithmic) and *p*-value (*y*-axis, logarithmic) between term and preterm infant milk and gastric samples. Filled circles indicate the peptide was significantly different between preterm and term infants (*p* < 0.05), whereas the hollow circles are non-significant.

Forty and 180 of these bioactive peptides were present in at least 75% of the milk or gastric samples, respectively, and were compared between preterm and term infants (Figure 8). Of the 40 peptides identified in at least 75% of milk samples, 4 were significantly higher in preterm mother’s milk compared with term mothers’ milk (three were antimicrobial and one stimulated cell proliferation). One additional peptide with antimicrobial activity was only identified in preterm mothers’ milk. Of the 180 potentially bioactive peptides identified in more than 75% of infant gastric samples, 145 trended towards greater abundance in term infants than in preterm infants. One peptide with potential antimicrobial activity was significantly higher in preterm gastric contents, whereas 21 peptides were significantly higher in the gastric contents of term infants. Of these 21 peptides significantly higher in term gastric samples, 12 had antimicrobial activity, four stimulated cell proliferation, and four had ACE-inhibitory activity. Additionally, one peptide with antimicrobial activity was found only in preterm infants, whereas six peptides with ACE-inhibitory and four peptides with antimicrobial activity were only found in term infants.

Of the eight peptides that were identified in all milk and gastric samples, human β-casein E^2^-K^18^ is 94% homologous with a known cell-proliferation-activating peptide [28] and human β-casein L^188^-V^211^ and L^189^-V^211^ have 89% and 85% homology, respectively, with a known antimicrobial peptide [29]. Human β-casein L^188-^V^211^was significantly higher in abundance in preterm milk compared with term milk, whereas in the gastric contents it was significantly higher in term infants compared with preterm infants.

## 4. Discussion

In this work, we examined the peptide profile of human milk and infant gastric samples collected from preterm-delivering and term-delivering mother–infant pairs. Peptidomics enables determination of specifically how proteins are digested within in vivo samples. Peptidomics has previously been applied to identify milk peptides in human milk [16,30,31], infant formula [32], preterm infant gastric samples [13,14,15], and infant stool samples [17]. However, to date, no peptidomics studies have been performed to compare milk protein digestion and the release of bioactive peptides within the stomachs of term and preterm infants.

The initial protein concentration in the human milk before fortification was not different between preterm and term infant mothers’ milk. The gastric protein concentrations were lower than what the summed protein concentration of the human milk and added fortification would be, which was likely due to digestion and dilution with gastric fluids in the stomach. These results align with a previous study [33]. The pH values of the gastric contents aligned with our previous study [7]. Although other previous studies have reported lower gastric pH values for infants of these age ranges and differences in pH between infants of different gestational age [4,34], these studies did not take gastric pH measurements after enteral milk feedings. The buffering capacity of milk proteins and salts present in the aspirates could explain the increased and consistent pH ranges for these infants.

The Orbitrap Fusion Lumos mass spectrometer used in this study allowed identification of a large number of peptides in each sample. The peptide profiles were more comprehensive than in a previous study of infant gastric digestion [14], which provides more complete data as basis for conclusion delivered by this study. The abundance of peptides identified in preterm-delivering mothers’ milk tended to be higher than that in term-delivering mothers’ milk, and, on an individual protein level, we observed a higher count and abundance of α_s1_-casein peptides in preterm mothers’ milk compared with term milk. In the first few weeks of life, the milk of preterm-delivering mothers is more nutrient-rich than that of term-delivering mothers [35]. The increased abundance of peptides could be related to the increased protein content in preterm milk.

The total count and abundance of peptides increased significantly and the average amino acid length of peptides decreased from mothers’ milk to infant gastric contents in both preterm and term infants. Peptide abundance was significantly higher in term infant gastric samples than in those of preterm infants. This finding indicates a higher break-down of protein in the term infant’s stomach compared with preterm infants and conforms with our previous data showing that preterm infants have lower protease activity in the stomach compared with term infants [7]. However, it should be noted that peptides were only identified in gastric samples collected 2 h after the initiation of feeding. In preterm infants, peptides continue to be released in the stomach up to 3 h after feeding [15]. There may be differences in the rate of gastric digestion and gastric emptying between preterm and term infants such that results may be different in gastric samples collected at a shorter or longer time after feeding.

Interestingly, we did not detect significant amounts of peptides from bovine proteins in the milk samples, however a large number of peptides from bovine β-lactoglobulin were present in the stomach, likely from the fortification of the milk prior to feeding. The large amount of bovine β-lactoglobulin peptides is counter-intuitive as previous in vitro studies have demonstrated that bovine β-lactoglobulin is partly resistant to digestion by gastric pepsin [36,37]. This peptidomics approach, however, does not allow determination of the remaining concentration of intact β-lactoglobulin.

The amino acid cleavage site-based enzyme analysis showed that the major cleavage site was after leucine in the gastric samples. These findings are logical as pepsin is the main gastric protease. Cathepsin D is present but inactive in milk and could be activated by the low pH of the stomach. The abundance of peptides resulting from cleavage after a leucine was significantly higher in the gastric samples of term infants than in preterm infants. This finding provides supporting evidence for the hypothesis that pepsin activity is higher in the stomachs of term infants compared with preterm infants, which was previously indicated by enzyme activity assays of gastric aspirates of preterm and term infants [7]. The proposed increased enzyme activity of pepsin in the term infant stomach compared with the preterm infant stomach supports the increased abundance of peptides identified in the term infant gastric samples compared with preterm infants’ gastric samples. This study is one of few studies comparing preterm and term digestion. The result reveals that preterm infants have lower digestive capacity than term infants, and therefore may have a limited ability to break-down milk proteins for nutrient absorption.

The differences in the peptide profiles of milk and gastric samples from either preterm or term infants could indicate a corresponding difference in the release of bioactive peptides. Therefore, we compared the identified peptides with the comprehensive database of known bioactive peptides derived from milk proteins (Milk Bioactive Peptide Database, MBPDB). Twenty-one biologically active peptides were significantly higher in term infants’ gastric samples, compared with only one peptide that was significantly higher in the preterm infant gastric samples. Though by far the most common functional attribution of milk peptides is ACE-inhibitory [26], the dominant bioactivity of peptides that were significantly higher in term infant gastric contents compared with preterm was antimicrobial activity. Thus, despite receiving a higher initial abundance of peptides in their milk, preterm infants have reduced total and antimicrobial peptide release in the stomach. The lower gastric peptide release seen in this study could be partially responsible for preterm infants’ slower growth and increased risk of infection. An initial decrease of peptides in the stomach could put the infants at a disadvantage by the time the feed reaches the intestine, where amino acids are absorbed and antimicrobial peptides are able to have effect [38]. The inclusion of proteases or supplemental bioactive milk peptides to the feed could compensate for the reduced digestion capacity of the preterm infant and should be studied further. Our study did not include identification of short peptides (2–5 amino acids long). As a high number of the known bioactive peptides are within this amino acid length range [39], potentially more bioactive peptides are present in mothers’ milk and infant gastric samples than are reported herein.

A limitation of this study is that term infant samples were collected from infants whose health circumstances required nasogastric tubes. Though these conditions are not known to affect digestion, whether the results from term infants are generalizable to the healthy term infant population is unknown.

## 5. Conclusions

This research contributes to a better understanding of gastric digestion of human and bovine milk proteins in preterm and term infants, an essential step in understanding overall protein digestion in these infants. We found an increase in peptide count and abundance and a decrease in amino acid length of peptides from mother’s milk to infant gastric contents. The overall higher abundance of peptides in term infant gastric contents compared with preterm infant gastric contents indicates a higher digestive capacity in term infants compared with preterm infants. Most individual proteins showed higher digestion in term gastric contents compared with preterm, and eleven of these proteins were significantly more digested, including human Ig gamma and bovine osteopontin. The lower level of pepsin/cathepsin D cleavage sites in the peptidomics data indicates that the apparent lower degree of protein digestion in preterm infants could be due to a lower gastric pepsin or cathepsin D activity compared with term infants. The apparent lower proteolytic activity in the preterm infant stomach resulted in a lower number of bioactive peptides, and particularly antimicrobial peptides, in their gastric samples compared with term infants. The lower level of antimicrobial peptides in the preterm infant stomach could represent lesser protection against pathogenic microbes than in term infants.

## Figures and Tables

**Table 1:** Demographic information for the mother–infant pairs sampled for milk and gastric contents. Data are presented as mean ± standard error (range).

Age/Weight	Preterm (*n =* 9)	Term (*n =* 4)
Gestational age at birth, weeks	26.8 ± 1.0 (24–32)	39.0 ± 0.5 (38–40)
Postnatal age at collection, day	24.6 ± 3.8 (8–41)	25.0 ± 6.5 (14–42)
Birth weight, g	930.6 ± 106.5 (620–1610)	3717.8 ± 119.3 (3360–3837)
Mother’s age, year	31.6 ± 1.9 (18–40)	20.8 ± 2.8 (18–29)

**Table 2:** Proteins with the highest number of identified peptides ^1^.

Protein	Count in Milk ^2^		Count in Gastric Contents ^3^	
	Preterm	Term	Preterm	Term
Bovine β-casein			197 ± 9	249 ± 18
Human β-casein	286 ± 27	228 ± 34	348 ± 28	308 ± 6
Human osteopontin	127 ± 22	130 ± 16	66 ± 9	58 ± 9
Bovine α_s1_-casein			132 ± 8	136 ± 14
Bovine κ-casein			109 ± 5	120 ± 18
Human α_s1_-casein	77 ± 8 *	49 ± 8	106 ± 11	75 ± 8
Human polymeric immunoglobulin receptor	84 ± 5 *	64 ± 6	67 ± 3	58 ± 6
Human lactoferrin	0 ± 0	1 ± 1	80 ± 10 *	41 ± 5
Bovine α_s2_-casein			45 ± 4	50 ± 4
Bovine β-lactoglobulin			63 ± 8	82 ± 9

^1^ Results are shown as mean ± standard error. Asterisks (*) indicate significant differences between preterm and term infants (*p* < 0.05); ^2^ Unfortified human milk; ^3^ Preterm infants were fed human milk fortified with bovine-based human milk fortifier and term infants were fed human milk fortified with high-calorie infant formula.
